# Effects of force magnitude on dental arches in cervical headgear therapy

**DOI:** 10.1093/ejo/cjab051

**Published:** 2021-08-09

**Authors:** Tuula Talvitie, Mika Helminen, Susanna Karsila, Pertti Pirttiniemi, Luca Signorelli, Reeta Varho, Timo Peltomäki

**Affiliations:** Vaasa Social Services and Health Care Division, Dental Service, Vaasa, Finland; Faculty of Health Sciences, Institute of Dentistry, University of Eastern Finland, Kuopio, Finland; Tays Research Services, Tampere University Hospital, Finland; Faculty of Social Sciences, Health Sciences, Tampere University, Finland; Turku Municipal Health Care Services, Dental Teaching Unit, Turku, Finland; Department of Oral Development and Orthodontics, Research Unit of Oral Health Sciences, University of Oulu, Finland; Medical Research Center, Oulu University Hospital, Finland; Private Practice, Wil, Switzerland; Turku Municipal Health Care Services, Dental Teaching Unit, Turku, Finland; Faculty of Health Sciences, Institute of Dentistry, University of Eastern Finland, Kuopio, Finland; Faculty of Medicine and Health Technology, Tampere University, Finland

## Abstract

**Aim:**

To study the influence of different force magnitudes on dental arches in cervical headgear (CHG) treatment.

**Material and methods:**

In this controlled clinical trial, patients (*n* = 40) were treated with CHG with light (L, 300 g, *n* = 22) or heavy force (H, 500 g, *n* = 18) magnitude. Subjects were asked to use CHG for 10 hours a day for 10 months. The outer bow of the CHG facebow was raised 10–20 degrees and the inner bow expanded 3–4 mm. Adherence to instructions and force magnitude were monitored with an electronic module (Smartgear, Swissorthodontics, Switzerland). Impressions for study models were taken before (T1) and after (T2) treatment and the study models were scanned into digital form (3Shape, R700 Scanner, Denmark). Measurements were made using the digital models (Planmeca Romexis, Model analyser, Finland).

**Results:**

During the treatment (T1–T2) the upper inter-canine distance increased by 2.83 mm (*P* = 0.000) and 2.60 mm (*P* = 0.000) in the L and H force magnitude groups, respectively. Upper inter-molar width increased by 3.16 mm (*P* = 0.000) and 2.50 mm (*P* = 0.000) in the L and H groups, respectively. Maxillary total arch perimeter increased by 6.39 mm (*P* = 0.001) and 6.68 mm (*P* = 0.001) in the L and H groups, respectively. In the amount of change over time, T1–T2, in the upper arch measurements, no significant difference was found between the groups. Lower inter-canine width increased by 0.94 mm (*P* = 0.005) and 1.16 mm (*P* = 0.000) in the L and H groups, respectively; no difference between the groups. Lower inter-molar distance increased by 2.17 mm (*P* = 0.000) and 1.11 mm (*P* = 0.008) in the L and H groups, respectively. At the end of the study, upper and lower inter-molar width was larger in the L group than in the H group (*P* = 0.039 and *P* = 0.022, respectively).

**Conclusion:**

CHG therapy is an effective method for expanding and releasing moderate crowding of the upper dental arch. The lower arch spontaneously follows the upper arch in widening effects, and minor expansion can also be seen on the lower arch. In the L group, larger inter-molar width was achieved on the upper and lower arch; probably due to better adherence to instructions. Light force is recommended for use in CHG therapy.

## Introduction

Signs of development of Class II malocclusion can be seen already at the primary dental stage, the traits of which are transferred to early mixed and permanent dentition. Self-correction during dentofacial growth and development is not to be expected without therapy (1–6). Class II molar relationship is associated with high variation in sagittal and vertical combinations of skeletal disharmony (7); most commonly associated with a retrusive mandibular position combined with a normal or protrusive or retrusive maxillary position and excessive lower face height (2, 7). Additionally, transversal discrepancy with a narrow upper dental arch is stated to be closely associated with Class II (2, 3, 6, 8–10), which can be detected at early age in the primary dentition and is maintained or can even worsen during development of the permanent dentition (2, 6, 11).

In general, more than 3 mm crowding in the early mixed dentition is found about 10 and 15 per cent in the upper and lower arch, respectively (12). Over 2 mm crowding in the mixed dentition is in most cases transferred into the permanent dentition (13). Less than 2 mm crowding in the mandibular anterior region in the early mixed dentition is thought to be related to a normal development stage and expected to self-correct during transition into the permanent dentition (14). Early expansion of the maxillary dental arch can release crowding, meaning less fixed appliance treatment and less extractions are needed in the later development stage (15).

Headgear (HG) is still widely used (16, 17) and is considered to be an effective orthodontic appliance in Class II therapy (18, 19). Like all removable orthodontic devices, HG is effective only when used in strict adherence to instructions throughout the HG therapy. Lack of compliance is one of the most important reasons not to use HG in Class II treatment (19).

In cervical headgear (CHG) activation for treatment numerous variations and combinations have been used. Facebow settings alone can fluctuate greatly. Expansion of the facebow inner arch can vary between 0 and 10 mm (20–27) and the long outer bow can be raised 0–20 degrees to prevent distal tipping of the first molars (22, 26–29). Used force magnitude can fluctuate from very light to very heavy (200–1000 g) depending on the target outcome (22, 25, 26, 30–34). Moreover, recommended CHG wear time varies greatly from 8 to 16 hours per day to up to 24/7 use (21, 22, 28, 33, 35–39).

Upper dental arch expansion has been found to result in spontaneous change in mandibular position (2, 8, 21, 27), which can simplify future treatment need and therapy arrangements (15, 20). In Class II therapy, correction of transversal discrepancy to improve sagittal relations is often needed. CHG therapy can influence sagittal and transversal planes simultaneously. With an expanded inner arch of the facebow, maxillary dental arch widening is achieved and considered to be effective and stable (9, 15, 18, 20, 22, 27, 40, 41). In addition, simultaneous mandibular dental arch widening and lengthening has also been reported (9, 22, 27, 41).

Maxillary displacement in the anterior direction can be restricted with HG (18, 22, 30, 42, 43) while at the same time changing the position of the maxillary first molars in the distal direction (18, 22, 30, 43) and allowing normal mandible development to continue (27, 43–45). Force magnitude has been postulated to be a decisive factor in achieving desired skeletal or dental improvements in CHG therapy. Less than 450 g force is thought to cause primarily dental effects and over 450 g to cause skeletal effects, the latter presumably due to exceeding the tooth moving threshold (46–48). Activated force magnitude in CHG therapy can be set to a certain level, light or heavy, but not to a specific force, because force fluctuation has been found to occur constantly during use (43). Despite different amounts of activated force, no pure dental or skeletal effects have been found in CHG use (43). The aim of this controlled clinical investigation was to study the effect of inner arch expansion of the facebow on the upper and lower dental arches when different force magnitudes in CHG treatment were used.

## Subjects and methods

The subjects and methods have been presented in our previous studies (43, 49, 50) but for clarification they are also presented here. The study subjects were recruited from a pool of children appropriate for the orthodontic treatment at the Health Care Center of Turku, in Turku, Finland. Possible study subjects were considered during screening if they met the following dental criteria:

1. Class II (or end-to-end) molar relationship
2. Mixed dentition stage
3. Moderate dental crowding

Complete orthodontic examination was accomplished with panoramic and cephalometric radiographs, dental and facial photos, and study model impressions. The plan for the orthodontic therapy was made by an experienced orthodontist (SK). The subjects were invited to take part in the study if the therapy method included only CHG. Subjects were to be treated as a part of a training course in orthodontics (clinical skills) at the Institute of Dentistry, University of Turku. The aim was to have one CHG patient per student, totalling at least 44 subjects. A pre-study power analysis was also conducted to determine an appropriate sample size for the investigation. Based on the findings of a previous study, it was estimated that a 3 mm difference in upper dental arch length gain over 12 months of CHG use could be expected (22). It was further assumed that the standard deviation (SD) would be the same, that is, 3 mm, in the light and heavy force magnitude groups. To achieve 80 per cent statistical power with a 5 per cent significance criterion, 17 subjects would be needed per group.

Approval for the research plan was acquired from the Ethical Committee of the Hospital District of Southwest Finland (ETMK: 77/180/2011). The invited children and their guardians signed an informed consent before booking an appointment with a dental student to fit the CHG. All stages of treatment were supervised closely by a senior orthodontist (SK) and a postgraduate orthodontic student (RV). The inner bow of the CHG was expanded (3–4 mm) and the long outer bow bent 10–20 degrees upwards in relation to the inner bow. The subjects were allocated into two groups, light (L) and heavy (H), with an activated force magnitude of 300 and 500 g, respectively. Force magnitude was controlled with an electronic module (Smartgear, Swissorthodontics, Switzerland) while the patient was sitting and looking straight ahead. The first 22 children who had booked appointment to start CHG treatment all received a CHG with light force module, as only light force modules were available when the clinical course for the dental students started.

**Figure 1:**
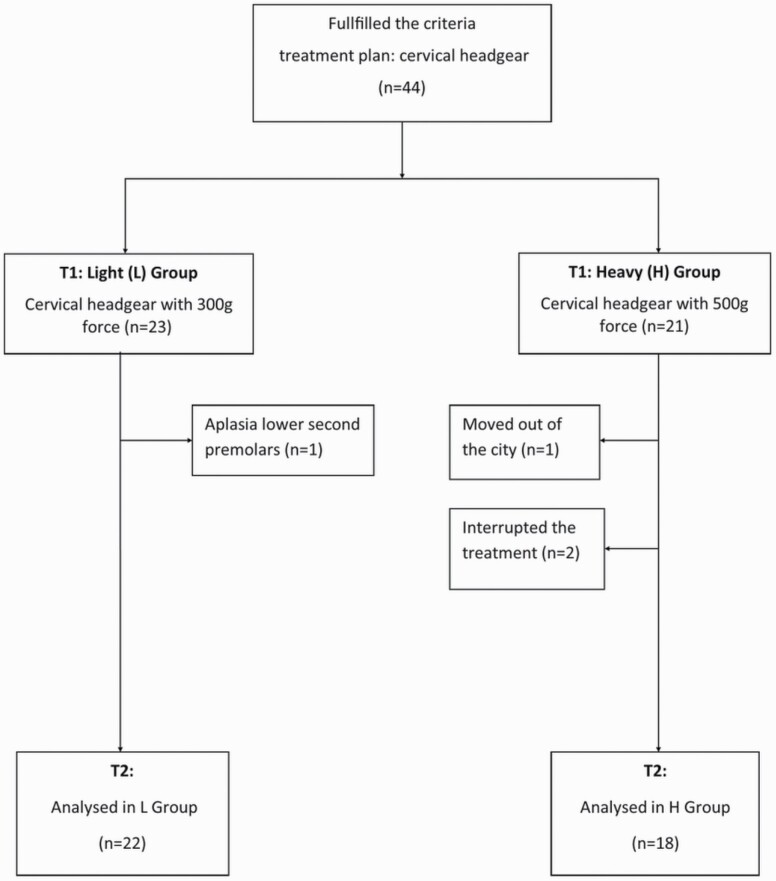
Chart illustrating the flow of the subjects T1–T2.

The children were instructed to wear the CHG for 10 hours a day, i.e. while sleeping, but the importance of wearing the HG in the early evening hours was emphasized. Patients visited the dental clinic every 6–8 weeks until the end of the study at 10 months. The use and force magnitude of the HG were controlled and readjusted during the appointments. The children and their parents did not know which group, L or H, they were assigned to but were fully informed and aware that they were taking part in the CHG study. At start, 44 patients were recruited for the study; two patients subsequently left the treatment programme: one moved out of the city, and one dropped out due to aplasia in lower permanent premolars noticed after the treatment (Figure 1). This trial is based on 40 children, 22 in the L group (8 males, 14 females) and 18 in the H group (7 males, 11 females). An adjustment period with 300 g force was carried out in the first 6–8 weeks for both groups in order to learn how to put on the CHG and to sleep with the device. The total number of days monitored was 11 344 over 10 months.

An electronic module (Smartgear, Swissorthodontics, Switzerland) was integrated into the neck strap on the right side to evaluate the hours of use and force magnitude. For safety reasons, a snap-away spring mechanism was used on both sides. The temperature and activated force magnitude were measured by the module once a minute and average values were calculated every 15 minutes. Accuracy of the temperature and force measurements was 1°C and 10 g, respectively. The module also allowed the time and date of CHG insertion/removal to be recorded. The information was read by the Smartgear Compliance Control System Version 2.1.2 (Swissorthodontics, Switzerland) and converted into table format (Excel, Windows 7, Microsoft) for analysis.

**Figure 2.**
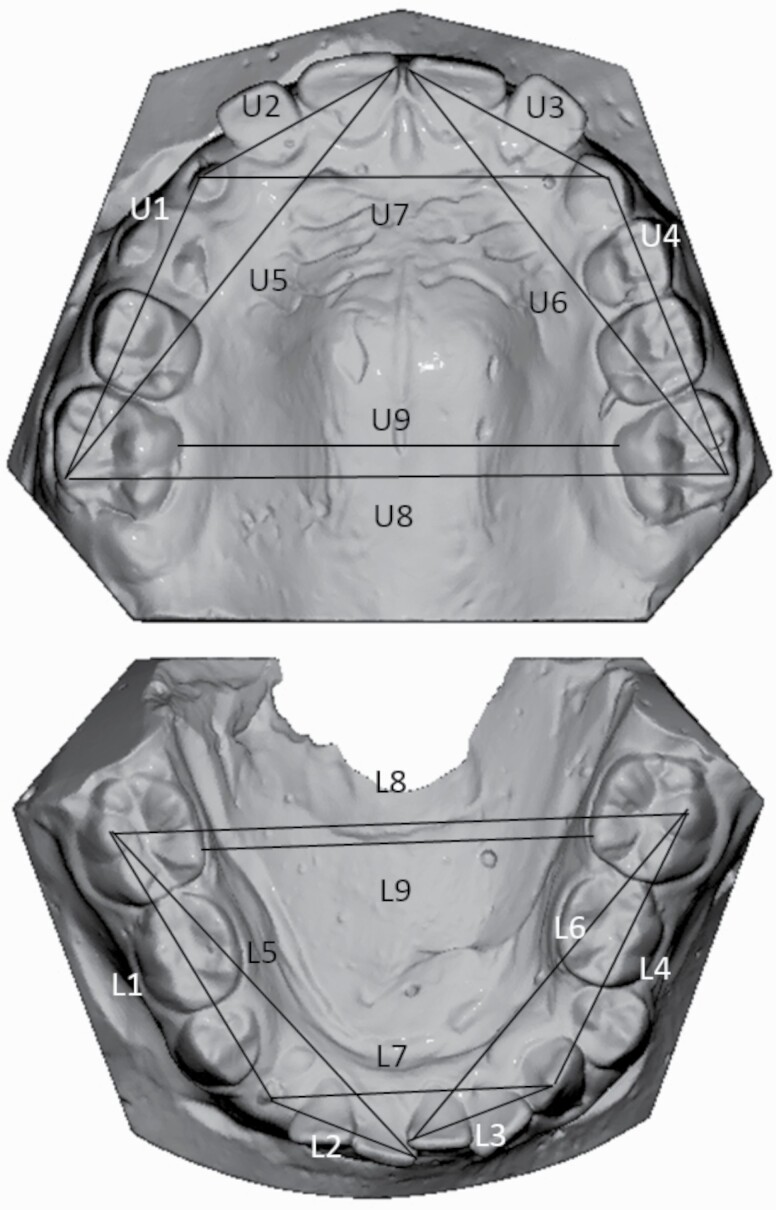
Distances (mm) on dental arches and are measured on 3D models digitally. Upper arch—U1 and U4: from the first permanent maxillary molar distobuccal cusp to the canine crown tip on same side. U2 and U3: from the canine crown tip to the incisor’s most mesial point on the same side. U5 and U6: from the first permanent maxillary molar distobuccal cusp tip to the incisor’s most mesial point on the same side. U7: from the canine to the canine crown tip on the upper arch. U8: from the permanent maxillary first molar distobuccal cusp to the permanent maxillary first molar distobuccal cusp on the contralateral side. U9: The shortest distance on the first permanent molars’ palatal surfaces on the upper arch. Lower arch—L1 and L4: from the permanent mandibular first molar distobuccal cusp to the canine crown tip on the same side. L2 and L3: from the canine crown tip to the incisor’s most mesial point on the same side. L5 and L6: from the permanent mandibular first molar distobuccal cusp to the incisor’s most mesial point on the same side. L7: from the canine crown tip to the canine crown tip on the lower arch. L8: from the permanent mandibular first molar distobuccal cusp tip to the permanent mandibular first molar distobuccal cusp on the contralateral side. L9: the shortest distance on the permanent first molars’ lingual surfaces on the lower arch.

The study models were scanned with a 3D scanner (3Shape, R700 Scanner, Denmark) and model analysing software (Romexis Model Analyser, Planmeca, Finland) was used to study the digital models. Linear measurements are described in Figure 2 (27). Measurements were made before (T1) and after the treatment (T2) by one investigator (TT) to describe the arch length and width in the posterior and anterior areas in the digital models. Blinding was ensured so that the examiner (TT) did not know which group the subject belonged to. Re-measurements of 10 pairs of pre- and post-treatment digital models were carried out at intervals of at least 2 weeks to measure the reliability of the measurements.

Inter-molar distance (U8 and L8) was measured from the first molar distobuccal cusp tip to the first molar distobuccal cusp tip on the contralateral side in both dental arches. Inter-molar distance (U9 and L9) was measured from the gingival margin to gingival margin the first molars. The inter-canine distance (U7 and L7) was measured from the cusp tip to the cusp tip on the contralateral side. Measurement was made from the primary canine tip if it was present or from the permanent canine tip if erupted sufficiently to enable measurement. If both primary and permanent canines were missing the measurement was made from the middle of the alveolar process. Leeway space was not taken into account in the transversal measurements.

### Statistical analysis

The results of linear measurements of the dental arches between the groups (L and H) at time points T1, T2, and T1–T2 were analysed with the Mann–Whitney *U*-test. Wilcoxon test was used to evaluate the change over time (T1–T2) within the group (L or H). *P*-values were considered statistically significant if they were less than 0.05. Reliability of the measurements was tested by intraclass correlation coefficients (ICCs) to compare re-measurements.

## Results

ICC values in the maxillary measurements (U1–U9) varied from 0.90 to 0.98; the highest value was in U2 and the lowest in U9. In the mandibular measurements (L1–L9) ICC varied from 0.84 to 0.98. L3 measurement had the lowest and L1 the highest ICC value. The values indicate satisfactory to excellent level and reliable repeatability of the measurements.

At beginning of the therapy, no age difference between the groups was identified. The mean age at T1 was 9.7 years (SD 0.73 years) and 9.9 years (SD 0.74 years) in the L and H groups, respectively.

At T1, four (22.2 per cent) subjects in the H group had one lower primary second molar exfoliated, while in the L group all lower primary second molars were *in situ*. At T2, five (22.7 per cent) subjects in the L group had lost the lower primary second molar on one side and 17 (77.3 per cent) subjects had lower primary second molars *in situ* on both sides; in the H group six subjects (33.3 per cent) had lost the lower primary second molar on both sides and three (16.7 per cent) on one side, and both were *in situ* in nine cases (50.0 per cent).

In 15 cases, Class I molar relationship on both sides was achieved during the treatment. In 21 subjects, Class I on one side was detected, and on the other side cusp-to-cusp or Class III molar relationship in 16 and 5 subjects, respectively. Even Class III molar relationship on both sides was found in two cases. Only in two cases Class II/Class II or Class II/ cusp-to-cusp molar relationship was found after the treatment.

### Upper dental arch

Measurements of the upper dental arch are presented in Table 1. At T1 some statistically significant difference was found between the groups. Measurements on the right side (U1 and U5) were larger in the L than in the H group (*P* = 0.034 and *P* = 0.034), respectively. Total arch perimeter (U1–U4) was larger in the L group than in the H group (*P* = 0.048).

**Table 1.** During the treatment enlargement was seen in measurements U1–U9 in both groups.

		Light force, L (300 g)							Heavy force, H (500 g)							L versus H	L versus H	L versus H
		T1		T2		T1–T2		P*	T1		T2		T1–T2		P*	T1	T2	T1–T2
*n* = 40	Valid	22		22		22		22	18		18		18		18	40	40	40
		Mean	SD	Mean	SD	Mean	SD		Mean	SD	Mean	SD	Mean	SD		*P*	*P*	*P*
U1 (mm)		27.58	1.29	29.34	2.12	1.76	1.80	**0.000**	26.34	1.99	29.05	2.34	2.71	2.05	**0.000**	**0.034**	0.600	0.153
U2 (mm)		17.79	1.76	19.13	1.53	1.33	0.80	**0.000**	17.44	1.40	18.44	0.97	1.00	0.90	**0.000**	0.563	0.100	0.366
U3 (mm)		18.29	1.43	19.48	1.38	1.19	0.86	**0.000**	17.73	1.31	18.89	0.93	1.16	1.11	**0.000**	0.262	0.163	0.657
U4 (mm)		27.29	1.43	29.40	2.00	2.11	1.29	**0.000**	26.60	1.59	28.40	1.69	1.80	1.30	**0.000**	0.271	**0.039**	0.427
U5 (mm)		43.26	2.40	45.54	2.69	2.28	1.70	**0.000**	41.38	2.64	44.59	2.80	3.21	1.34	**0.000**	**0.034**	0.209	0.147
U6 (mm)		43.38	2.38	46.06	2.55	2.68	1.33	**0.000**	41.78	2.24	44.26	2.17	2.48	1.17	**0.000**	0.112	**0.040**	0.638
U7 (mm)		31.22	1.80	34.04	2.46	2.83	1.74	**0.000**	30.90	2.24	33.50	1.76	2.60	1.87	**0.000**	0.677	0.443	0.717
U8 (mm)		52.00	2.41	55.17	2.37	3.16	1.64	**0.000**	51.04	3.03	53.54	2.37	2.50	1.78	**0.000**	0.312	**0.039**	0.199
U9 (mm)		32.29	2.30	35.92	2.55	3.63	1.93	**0.000**	31.76	2.39	34.42	1.70	2.66	1.68	**0.000**	0.427	**0.020**	0.075
U1–U4 (mm) Total arch perimeter		90.96	4.39	97.35	5.81	6.39	3.17	**0.001**	88.12	4.62	94.79	4.92	6.68	3.13	**0.001**	**0.048**	0.125	0.840

At T1 and T2 some difference was seen between the groups, but the change T1–T2 was similar in both groups. P* = Wilcoxon test; *P* = Mann–Whitney *U*-test.

**Figure 3.**
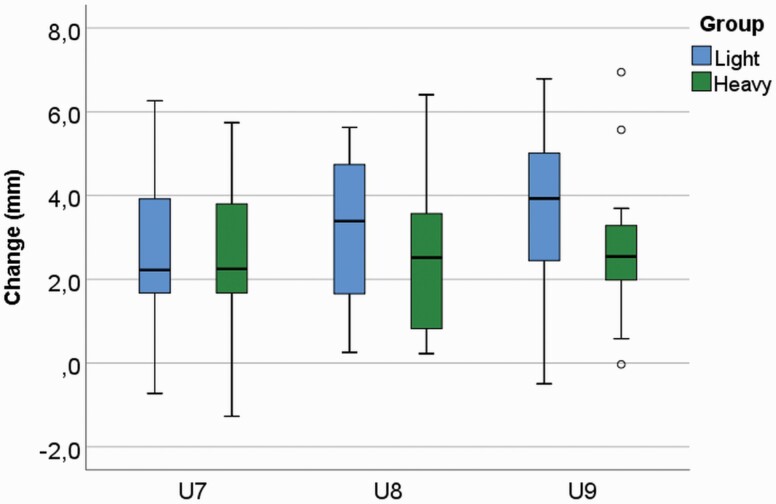
Cervical headgear (CHG) was effective with light and heavy force to enhance the maxillary inter-canine (U7) and inter-molar (U8 and U9) distance during the treatment, with great individual variability; U7: *P* < 0.001 and *P* < 0.001; U8: *P* < 0.001 and *P* < 0.001; U9: *P* <0.001 and *P* < 0.001; in the L and H groups, respectively.

**Figure 4.**
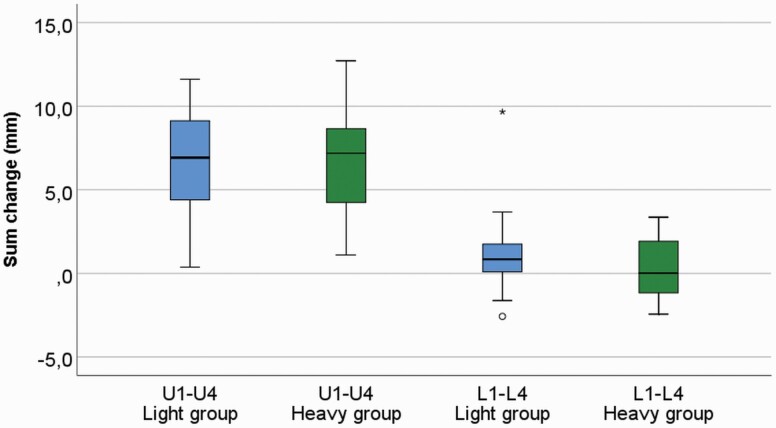
The total arch perimeter in the upper arch (U1–U4) increased over 6 mm in both groups during cervical headgear (CHG) therapy, with great individual variability (*P* = 0.001 and *P* = 0.001 in the L and H group, respectively). In the lower dental arch, enhancement in the total arch perimeter was seen (L1–L4) in the L group (*P* = 0.010); in the H group, no change was seen during the treatment.

Statistically significant increase was found in all measurements in the maxillary dental arch in both groups during the CHG therapy, with great individual variability. In the amount of change over time, T1–T2, in the upper arch measurements, no significant difference was seen between the groups. Inter-canine (U7) width increased 2.83 mm (*P* < 0.001) and 2.60 mm (*P* < 0.001) and inter-molar width (U8) 3.16 mm (*P* < 0.001) and 2.50 mm (*P* < 0.001), and (U9) 3.63 mm (*P* < 0.001) and 2.66 mm (*P* < 0.001) in the L and H groups, respectively (Figure 3). Lateral segments were found to lengthen as follows—U1: 1.76 mm (*P* < 0.001) and 2.71 mm (*P* < 0.001) and U4: 2.11 mm (*P* < 0.001) and 1.80 mm (*P* < 0.001) in the L and H groups, respectively. Increases in the anterior segment were U2: 1.33 mm (*P* < 0.001) and 1.00 mm (*P* < 0.001) and U3: 1.19 mm (*P* < 0.001) and 1.16 mm (*P* < 0.001) in the L and H groups, respectively. Sagittal arch length (U5 and U6) increased in the L group by 2.28 mm (*P* < 0.001) and 2.68 mm (*P* < 0.001), respectively, and in the H group by 3.21 mm (*P* < 0.001) and 2.48 mm (*P* < 0.001), respectively. Total arch perimeter (U1–U4) was statistically significantly larger in the L group at T1: 90.96 and 88.12 mm (*P* = 0.048) but not at T2: 97.35 and 94.79 mm (*P* = 0.125) in the L and H groups, respectively; during T1–T2 the increase was 6.39 mm (*P* = 0.001) and 6.68 mm (*P* = 0.001) in the L and H groups, respectively (Figure 4).

Analogous to the beginning of the study, some statistically significant differences were found between the groups at the end of the study. On the left side, U4 and U6 distances were larger in the L group (*P* = 0.039 and *P* = 0.040, respectively). Inter-molar distance was wider in the L group (U8: *P* = 0.039 and U9: *P* = 0.020).

### Lower dental arch

Measurements of the lower dental arch are presented in Table 2. At T1 distances L3, L5, L6, and L1–L4 were found to be statistically significantly different between the groups. The anterior segment (L3) was shorter in the H group (*P* = 0.015). Sagittal arch length measurement (L5 and L6) was shorter in the H group (*P* = 0.008 and *P* = 0.011, respectively). Total arch perimeter (L1–L4) was shorter in the H group (*P* = 0.004).

**Table 2.** Statistically significant enlargement was found in the lower dental arch during CHG therapy in the L and H group in most measurements, but the change was larger in the L group, particularly in inter-molar width.

		Light force, L (300 g)							Heavy force, H (500 g)							L versus H	L versus H	L versus H
		T1		T2		T1–T2		P*	T1		T2		T1–T2		P*	T1	T2	T1–T2
*n* = 40	Valid	22		22		22		22	18		18		18		18	40	40	40
		Mean	SD	Mean	SD	Mean	SD		Mean	SD	Mean	SD	Mean	SD		*P*	*P*	*P*
L1 (mm)		27.73	1.27	28.28	2.51	0.55	2.00	0.147	26.87	1.76	26.78	1.98	−0.08	0.69	0.799	0.070	**0.024**	0.229
L2 (mm)		13.86	0.96	14.15	0.89	0.29	0.61	0.068	13.14	1.34	13.47	1.13	0.32	0.51	**0.015**	0.100	0.096	0.804
L3 (mm)		14.11	0.94	14.39	0.67	0.28	0.65	**0.033**	13.26	1.20	14.00	1.15	0.74	0.84	**0.002**	**0.015**	0.120	0.120
L4 (mm)		27.67	1.34	27.70	1.26	0.03	0.99	0.799	26.89	2.28	26.13	2.31	−0.76	1.19	**0.014**	0.299	**0.004**	**0.034**
L5 (mm)		38.10	1.89	38.77	1.89	0.67	0.71	**0.000**	36.97	1.78	36.77	2.27	−0.20	0.80	0.304	**0.008**	**0.001**	**0.003**
L6 (mm)		38.55	1.69	38.91	1.48	0.35	0.89	0.068	37.07	1.96	36.89	2.27	−0.18	0.95	0.304	**0.011**	**0.000**	0.070
L7 (mm)		25.90	1.68	26.84	1.52	0.94	1.46	**0.005**	25.09	2.61	26.25	2.39	1.16	1.42	**0.000**	0.527	0.563	0.717
L8 (mm)		45.98	2.39	48.15	2.07	2.17	1.29	**0.000**	45.07	2.75	46.18	2.66	1.11	1.56	**0.008**	0.262	**0.022**	**0.032**
L9 (mm)		31.88	1.76	33.38	1.55	1.50	0.84	**0.000**	30.82	2.30	31.49	2.08	0.67	0.99	**0.007**	0.169	**0.004**	**0.003**
L1–L4 (mm) Total arch perimeter		83.36	3.24	84.52	4.04	1.16	2.38	**0.010**	80.16	3.97	80.37	4.14	0.21	1.69	0.734	**0.004**	**0.001**	0.299

P* = Wilcoxon test; *P* = Mann–Whitney *U*-test.

**Figure 5.**
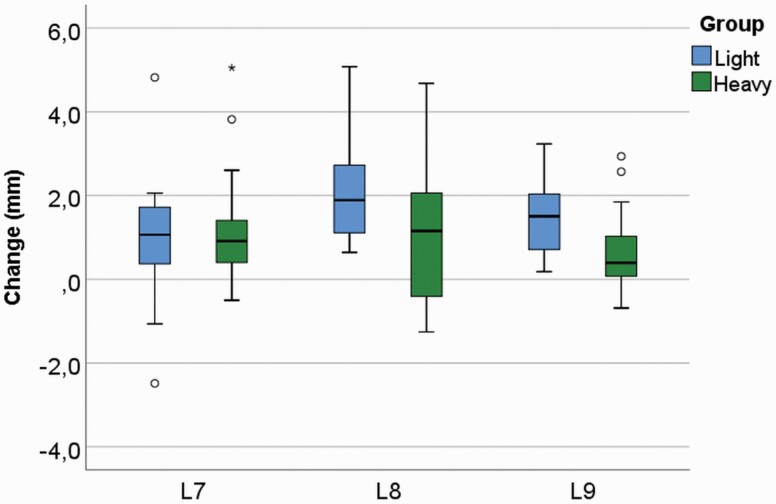
Inter-canine distance (L7) and inter-molar distance (L8 and L9) enhanced in the lower arch in the L and H groups during the cervical headgear (CHG) therapy (L7: *P* = 0.005 and *P* < 0.001; L8: *P* < 0.001 and *P* = 0.008; L9: *P* < 0.001 and *P* = 0.007; in the L and H groups, respectively). The change was larger in the molar area in the L group (*P* = 0.032 and 0.003 in L8 and L9, respectively).

During the treatment, T1–T2, an increase was seen in both groups in inter-canine (L7) distance (Figure 5): 0.94 mm (*P* = 0.005) and 1.16 mm (*P* < 0.001) in the L and H groups, respectively, with great individual variability. Widening of the inter-molar distance (L8 and L9) was found as follows—L8: 2.17 mm (*P* < 0.001) and 1.11 mm (*P* = 0.008) and L9: 1.50 mm (*P* < 0.001) and 0.67 mm (*P* = 0.007) in the L and H groups, respectively, with great individual variability. In the anterior segment (L2 and L3) slight, but statistically significant enlargement during the therapy was found in the H group: 0.32 mm (*P* = 0.015) and 0.74 mm (*P* = 0.002), respectively; in the L group a statistically significant minor increment was found only in L3: 0.28 mm (*P* = 0.033). A minor, but statistically significant increase in total arch perimeter (L1–L4) was found only in the L group: 1.16 mm (*P* = 0.010; Figure 4). The change during T1–T2 was larger in the L group in inter-molar width (L8 and L9; *P* = 0.032 and *P* = 0.003, respectively). In lateral segment (L4) and sagittal arch length (L5), the T1–T2 change was statistically different between groups (*P* = 0.034 and *P* = 0.003, respectively); in the L group a slight increment and in the H group a decrement were found.

At the end of the study, lateral segments were larger in the L group (L1 and L4, *P* = 0.024 and *P* = 0.004, respectively). Lower dental arch was narrower in the molar region (L8 and L9) in the H group (*P* = 0.022 and *P* = 0.004). Sagittal arch length (L5 and L6) was shorter in the H group (*P* = 0.001 and *P* < 0.001, respectively). Total arch perimeter (L1–L4) was shorter in the H group (*P* = 0.001), analogous to the difference before the therapy.

## Discussion

This controlled clinical investigation studied how different force magnitude influences dental arch dimensions during CHG therapy. Subjects were allocated into light (L) and heavy (H) groups and asked to wear CHG for 10 hours daily, emphasizing the importance of use during early evening hours. Hours used and force magnitude were monitored with an electronic module. The children were all in co-operative age, in pre-puberty (51). After a 10-month CHG treatment period, re-evaluation was made for a future treatment plan. A Class I molar relationship was achieved in most of the cases and treatment was continued with fixed appliances.

According to our previous study on adherence to instructions and fluctuation of force magnitude in CHG therapy (49), children in the L group used the device during the study period over 9 hours a day and in the H group for less than 8 hours. Force magnitude in CHG therapy can be set to a certain level, light or heavy, although the force magnitude fluctuates greatly and continually (0–900 g). Mean force magnitude during the CHG was approximately 320 and 460 g in the L and H groups, respectively, with high individual variability. In our cephalometric study (43), over 20 skeletal and dental measurements were evaluated before and after the treatment. According to the cephalometric measurements, the groups were homogenous at the start of the study. The only difference was inclination of the upper incisors, which were 6 degrees more proclined in the L than in the H group (43). In the present study, some differences were initially found in the dental arch measurements. This difference between the groups can be considered to be of dental origin based on the cephalometric analysis (43).

To the best of our knowledge, this is the first study to investigate how different force magnitude influences dental arches in CHG therapy. Based on this study, dental effects on the upper and lower dental arches can be achieved both with light and heavy force. Enhancement was seen in the transversal and sagittal dimensions on the maxillary arch in both groups. In the mandibular arch, minor, but mostly statistically significant changes in both groups were also found. After the treatment the mandibular inter-molar distance was larger in the L group; the change during the follow-up period was significantly larger in the L group.

In the HG therapy, expansion of the inner facebow arch can be applied up to 10 mm in order to broaden the upper dental arch while achieving at the same time as distal movement of the first molars and restriction of forward displacement of the maxilla (15, 25, 40). Widening of the maxillary molars has been found to lead to spontaneous buccal tipping of the mandibular molars, supposedly due to occlusal factors, and eventually to mild changes in the mandibular dental arch width (15, 20, 22, 27, 41, 52). This study is in agreement with the previous studies. Modest expansion (3–4 mm) of the inner facebow arch applied in the present study seems, however, to be clinically sufficient to increase the maxillary and mandibular inter-canine and inter-molar distance. Widening of the upper arch is probably partly due to the force magnitude loaded on the molars and partly due to the expanded facebow inner arch, which lifts the buccal muscles from the dental arch, allowing enlargement (25). Adherence to instructions has been found to be better with light force treatment (49), which may clarify why the upper and lower inter-molar distance was larger in the L group at the end of the treatment. In addition to the longer duration of the acting force, lower force magnitude itself has been found to lead to more favourable tooth movement (46–48).

Light and heavy force CHG can be used to gain more space in the anterior and posterior segments of the maxillary arch. Space gain in the upper arch has been found to be mainly due to the distal movement of the maxillary molars (25, 43, 53), seen as an increase in lateral segment dimensions (27). Furthermore, the inter-canine and inter-molar distance was increased and the maxillary incisors proclined in the labial direction (20, 43). The more than 6 mm increase found in total arch perimeter can release crowding, which in the following treatment phase may mean fewer permanent tooth extractions and less need for fixed appliance for tooth alignment (15). HG therapy can be considered a clinically important method for reducing mild and moderate crowding in the mixed dentition (25).

Mandibular dental arch length is known to decrease after age 8 during normal occlusal development (54, 55). During normal development, reduction of lower dental arch perimeter can be expected to be approximately 3 mm between 7 and 13 years (55, 56). Despite the reduction it is possible to see relieving of anterior crowding during development from the early mixed dentition to the early permanent dentition due to Leeway space in the lateral segments (57). Crowding in the lower arch can be relieved by tipping lower incisors labially, increasing the inter-canine distance, enhancing the mandibular arch width in the posterior area, and uprighting mandibular first molars (13, 58, 59). According to the present study, CHG is an effective method for maintaining or even increasing total lower arch perimeter (L1–L4). Based on our previous study (43), no change in lower incisor inclination was found during the study period. Enhancement in the sagittal arch length (L5 and L6) can be considered to be mostly from posterior origin, increase in the inter-molar width, and probably also the mandibular molar uprighting. In the H group, the dentition development stage was more advanced (more exfoliated lower primary second molars) than in the L group. Changes in the H group are suggested to be related to normal development from mixed dentition to early permanent dentition, which normally includes mesial migration of the mandibular first molars and canine movement into the Leeway space (56, 57, 60). We suggest that with CHG therapy normal reduction of lower dental arch perimeter can be alleviated or even increased by increasing inter-molar and inter-canine distance in the mixed dentition. Changes were seen in both groups, but in the L group the change was larger. Changes in the lower arch during the HG therapy are minor in absolute terms (mm) but in borderline cases can influence future treatment decisions, e.g. permanent tooth extractions (27). Expansion in the lower molar area is stated to be stable, but some relapse in inter-canine distance can be found at long-term follow-up (15, 61).

The maxillary molar movement in the distal direction is not all bodily movement, but tilting can also occur during HG therapy (23, 28, 29, 50). In our previous study, maxillary first and second molars were found have tipped more distally with heavy force than with light force (50). The follow-up period in this study was rather short, 10 months, and thus not all patients had early permanent dentition by the end of the study. After the treatment the upper first molars can be expected to migrate mesially and upright (23, 28, 62); consequently, some of the space gained in the arch may be lost during the post-treatment period.

### Strengths and limitations

Allocation of the children to the study groups occurred by booking a suitable appointment to start the CHG therapy and not by sealed-envelope randomization. Incomplete randomization is considered a significant limitation of the study, preventing it from being classified as a randomized clinical trial. It would have been appropriate to include a control group of the same age without treatment, but no ethical committee would have approved such a study plan despite the inclusion of radiographic examination. In addition, the follow-up time was too short to study the changes in irregularity and release of crowding on the dental arches, as not all subjects would have had early permanent dentition at the end of the study period.

The strengths of this investigation were that at start of the study the groups were homogenous, at same age with small range, skeletally similar according to the cephalometric analysis, of similar occlusal status (43), and all subjects were of co-operative age, in pre-puberty.

## Conclusion

1. CHG therapy with light or heavy force magnitude is an effective method to expand the upper dental arch. Inter-molar and inter-canine distance increased during the therapy in both groups.
2. Mild to moderate crowding on the upper dental arch can be released with CHG therapy, the total arch perimeter increased over 6 mm in both groups. Most of the space is achieved by distalizing the upper first molars.
3. Milder spontaneous widening was found on the lower arch during the CHG therapy in both groups.
4. Light force magnitude is recommended for use in CHG therapy; larger inter-molar width was achieved on the upper and lower arch; probably due to better adherence to instructions. Expanded facebow inner arch lifts the buccal muscles apart from the dental arch; the CHG has more time to effect in the L group.

## Funding

None to declare.

## Conflicts of interest

None to declare.

## Data availability

The data underlying this article cannot be shared publicly to protect the privacy of the individuals who participated in the study. The data will be shared on reasonable request to the corresponding author.
